# Supplementary material for: HIVprotI: an integrated web based platform for prediction and design of HIV proteins inhibitors
Source: J Cheminform. 2018 Mar 9;10:12. doi: 10.1186/s13321-018-0266-y (PMC5845081; doi:10.1186/s13321-018-0266-y)
Supplement: Supplementary file 2 — Additional file 2. Source code of HIVProtI web server. [file 13321_2018_266_MOESM2_ESM.zip › HIVprotI_Source-code/hivprotI/weka-3-6-6/doc/weka/clusterers/forOPTICSAndDBScan/OPTICS_GUI/ResultVectorTableModel.html]

ResultVectorTableModel


---


|  |  |  |
| --- | --- | --- |
| **Overview** \| **Package** \| **Class** \| **Tree** \| **Deprecated** \| **Index** \| **Help** \| **Weka's home** | |  |
| **PREV CLASS**   **NEXT CLASS** | **FRAMES**    **NO FRAMES**     **All Classes** |  |
| SUMMARY: NESTED \| FIELD \| CONSTR \| METHOD | DETAIL: FIELD \| CONSTR \| METHOD |  |


---


## weka.clusterers.forOPTICSAndDBScan.OPTICS\_GUI Class ResultVectorTableModel

```
java.lang.Object
  javax.swing.table.AbstractTableModel
      weka.clusterers.forOPTICSAndDBScan.OPTICS_GUI.ResultVectorTableModel
```

**All Implemented Interfaces:**: java.io.Serializable, javax.swing.table.TableModel, RevisionHandler

---

``` public class ResultVectorTableModel extends javax.swing.table.AbstractTableModel implements RevisionHandler ```

ResultVectorTableModel.java

**Version:**
:   $Revision: 1.4 $

**Author:**
:   Zhanna Melnikova-Albrecht (melnikov@cip.ifi.lmu.de), Rainer Holzmann (holzmann@cip.ifi.lmu.de)

**See Also:**: Serialized Form

---

**Constructor Summary**

`ResultVectorTableModel(FastVector resultVector)`
  
          Constructs a default `DefaultTableModel`
which is a table of zero columns and zero rows.


| **Method Summary** | |
| --- | --- |
| `int` | `getColumnCount()`             Returns the number of columns of this model. |
| `java.lang.String` | `getRevision()`             Returns the revision string. |
| `int` | `getRowCount()`             Returns the number of rows of this model. |
| `java.lang.Object` | `getValueAt(int row, int column)`             Returns the value for the JTable for a given position. |

**Methods inherited from class javax.swing.table.AbstractTableModel**

`addTableModelListener, findColumn, fireTableCellUpdated, fireTableChanged, fireTableDataChanged, fireTableRowsDeleted, fireTableRowsInserted, fireTableRowsUpdated, fireTableStructureChanged, getColumnClass, getColumnName, getListeners, getTableModelListeners, isCellEditable, removeTableModelListener, setValueAt`

**Methods inherited from class java.lang.Object**

`equals, getClass, hashCode, notify, notifyAll, toString, wait, wait, wait`

**Constructor Detail**

### ResultVectorTableModel

```
public ResultVectorTableModel(FastVector resultVector)
```

:   Constructs a default `DefaultTableModel`
    which is a table of zero columns and zero rows.


**Method Detail**

### getRowCount

```
public int getRowCount()
```

:   Returns the number of rows of this model.
    The number of rows is the number of dataObjects stored in the resultVector

    :   **Specified by:**: `getRowCount` in interface `javax.swing.table.TableModel`
    :   **Returns:**: the number of rows of this model

---


### getColumnCount

```
public int getColumnCount()
```

:   Returns the number of columns of this model.
    The number of columns is 4 (dataObject.key, dataobject, c\_dist, r\_dist)

    :   **Specified by:**: `getColumnCount` in interface `javax.swing.table.TableModel`
    :   **Returns:**: int The number of columns of this model

---


### getValueAt

```
public java.lang.Object getValueAt(int row,
                                   int column)
```

:   Returns the value for the JTable for a given position.

    :   **Specified by:**: `getValueAt` in interface `javax.swing.table.TableModel`
    :   **Parameters:**: `row` - The row of the value: `column` - The column of the value **Returns:**: value

---


### getRevision

```
public java.lang.String getRevision()
```

:   Returns the revision string.

    :   **Specified by:**: `getRevision` in interface `RevisionHandler`
    :   **Returns:**: the revision


---


|  |  |  |
| --- | --- | --- |
| **Overview** \| **Package** \| **Class** \| **Tree** \| **Deprecated** \| **Index** \| **Help** \| **Weka's home** | |  |
| **PREV CLASS**   **NEXT CLASS** | **FRAMES**    **NO FRAMES**     **All Classes** |  |
| SUMMARY: NESTED \| FIELD \| CONSTR \| METHOD | DETAIL: FIELD \| CONSTR \| METHOD |  |


---
